# GAN-Based Differential Private Image Privacy Protection Framework for the Internet of Multimedia Things

**DOI:** 10.3390/s21010058

**Published:** 2020-12-24

**Authors:** Jinao Yu, Hanyu Xue, Bo Liu, Yu Wang, Shibing Zhu, Ming Ding

**Affiliations:** 1 School of Space Information, Space Engineering University, Beijing 101416, China; yujinaosay@163.com (J.Y.); zhushibingsay@163.com (S.Z.); 2 School of Computer Science, University of Technology Sydney, Sydney, NSW 2007, Australia; Hanyu.Xue@student.uts.edu.au (H.X.); bo.liu@uts.edu.au (B.L.); 3 Institute of Artificial Intelligence and Blockchain, Guangzhou University, Guangzhou 510006, China; 4 Data61, CSIRO, Sydney, NSW 2015, Australia; Ming.Ding@data61.csiro.au

**Keywords:** Internet of Multimedia Things (IoMT), image privacy, object detection, deep learning, generative adversarial network, differential privacy

## Abstract

With the development of the Internet of Multimedia Things (IoMT), an increasing amount of image data is collected by various multimedia devices, such as smartphones, cameras, and drones. This massive number of images are widely used in each field of IoMT, which presents substantial challenges for privacy preservation. In this paper, we propose a new image privacy protection framework in an effort to protect the sensitive personal information contained in images collected by IoMT devices. We aim to use deep neural network techniques to identify the privacy-sensitive content in images, and then protect it with the synthetic content generated by generative adversarial networks (GANs) with differential privacy (DP). Our experiment results show that the proposed framework can effectively protect users’ privacy while maintaining image utility.

## 1. Introduction

**Figure 1:**
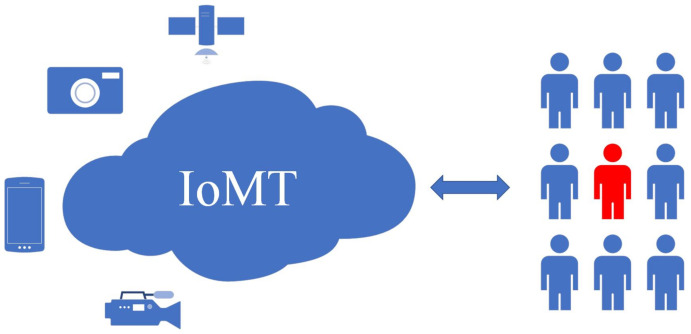
The IoMT collects sensitive private data through sensors (phones, cameras, drones, monitoring cameras) that might leak personal privacy.

The recent advances in multimedia recording devices, such as phones, cameras, drones, and other types of sensors, have greatly facilitated the collection of multimedia data, especially in the form of images and videos. In such an era of IoMT, a massive number of images are widely used, not only by social network personal users but also by government and companies. Image data is the most representative type of data in an IoMT data collection and can contain sensitive information that might be used to uncover personal information. The relationship between IoMT sensors (phones, cameras, drones, monitoring cameras) and personal private information shows in Figure 1.

Data mining attacks on images can easily cause privacy leakage, which can have serious consequences. The issue of privacy leakage has attracted the attention of the public in recent years and has aroused public concern about this issue. Moreover, privacy issues are no longer just personal concerns as many countries have launched privacy acts and laws. For example, the European General Data Protection Regulation (GDPR) took effect on 25 May 2018 [1]. Any violations of the regulation will trigger heavy fines and penalties. GDPR emphasizes the protection of “personal data”, interpreting this as “any information relating to an identified or identifiable natural person (‘data subject’); an identifiable natural person is one who can be identified, directly or indirectly, in particular by reference to an identifier such as a name, an identification number, location data, an online identifier or to one or more factors specific to the physical, physiological, genetic, mental, economic, cultural or social identity of that natural person” [2]. According to this definition, images include a variety of personal identifiers such as people’s faces, text and license plates. Therefore, effective image privacy protection techniques are in urgent need. The research community has expended considerable effort on image privacy protection. The early works mostly focus on the access control of the data, i.e., privacy protection by safeguarding against unauthorized access. This can be achieved by setting the preferences of users [3,4] or tag controls [5,6]. However, these methods cannot be applied to scenarios where images are shared openly, but some sensitive information needs to be concealed. For example, in the “Google Street View” application, we have full access to photos showing the streets while people’s faces and other personal identifiers have been obfuscated, e.g., by blurring. To achieve this, privacy protection methods need to detect, and then cover/remove/replace sensitive content in images. Several recent studies have explored this direction [7,8,9,10,11,12,13,14,15,16]. For example, Viola et al. [8] used a sliding window detector to identify and blur the license plates in Google Street View images. Yu et al. [11] used a deep multitask learning algorithm to detect privacy-sensitive objects and provide simple protection by blurring. Overall, most of the existing work undertakes personal data detection as the first step in privacy protection, relying on simple approaches such as blurring or pixelation. Consequently, the image utility suffers to a considerable extent. It not only makes the images look unnatural, but the person who looks at the image is aware that the obfuscated part is private. Moreover, such a protection mechanism is powerless against the emerging attacks based on advanced deep neural networks. For example, McPherson et al. [17] use artificial neural networks to recover hidden information from images protected by pixelation, blurring and P3. The method obtained good results on different data sets, MINIST 80%, CIFAR-10 75%, AT&T dataset 95%, FaceScurb 57%.

Moreover, the existing methods almost entirely focus on single object protection, such as face or text. However, most images that require privacy protection have multiple objects that need to be protected (For example, in street view images, human faces and license plates need to be protected at the same time).

Current methods are unable to find a way to quantify the tradeoff between image usability and privacy protection. To tackle this, we use DP to control the generation of de-identify objects in images to mitigate privacy threats.

To overcome these obstacles, we propose a three-stage framework for image privacy protection in this paper. The framework consists of three steps: (1) privacy-sensitive content detection and position extraction powered by a deep convolutional neural network: we use CNN networks to detect various objects in images and classify objects as either private or non-private; (2) projecting real private objects into latent space: we use generative adversarial networks (GANs) to project the real private objects of the images into latent space and obtain the corresponding latent vector $\omega^{*}$. (3) private content generation controlled by DP (de-identification): we use Laplace noise into the latent vector $\omega^{*}$ and to generate the de-identification content. Finally, we replace the originally private objects with the synthetic ones to protect users’ privacy.

In order to evaluate the performance of our proposed framework, we conduct extensive experiments on a real-world image data set collected by IoMT cameras, and investigate two types of personal identifier-related data: license plate and face. We choose these two types of objects as they represent the two most significant categories of personal identifiers in images.

In summary, the contributions of this paper are as follows:

- We propose an image privacy protection framework that can protect the privacy in IoMT images.
- We propose a GAN-based method to generate the replacement content for private objects in images.
- We use differential privacy methods to control image generation between image usability and privacy protection.

The remainder of the paper is organized as follows. In Section 2, we review the related work. In Section 3, we give the definition and foundation of the methods. In Section 4, we present our framework on multimedia privacy protection based on Mask-RCNN and synthetic content generation using GANs. In Section 5, we detail the experiment results of our framework for multiobject privacy protection (street view scenarios). In Section 6, we conclude the paper and outline future work.

## 2. Related Work

Privacy protection, in general, has been extensively studied in recent years. Of all the research in this area, differential privacy (DP) has attracted the most attention and has been applied to many different applications. Therefore, in this section, we review the most relevant research on image privacy and the related fundamental deep learning research, including: (1) image privacy issues and protection; (2) deep learning and object detection of images; (3) content generation; and (4) privacy protection.

### 2.1. Image Privacy Issues and Protection

Image privacy issues first attracted attention with the raipd development of social networks. The proliferation of social networks generated a massive number of photos flooding the Internet, some of which contain sensitive information. For example, Tang et al. [18] illustrated the problem of unpermitted photo sharing in social media and Pesce et al. [19] investigated the use of photo tags by third parties to compromise a user’s privacy. The image privacy issue becomes more severe with the widespread use of facial recognition systems, as people worry that their faces might be used by organizations for profiling or social control.

To combat image privacy attacks, the previous mainstream method uses access control on sensitive content. Mannan et al. [3] use instant messaging (IM) networks to control personal web content sharing. Vyas et al. [4] use annotation data to predict the privacy preferences of users and control shared content. Wang et al. [5] studied privacy control on Facebook, and Squicciarini et al. [6] proposed collaborative privacy management to enable users to collaboratively control their photos. Similarly, to deal with the privacy issue in facial recognition systems, the current countermeasure is simply banning [20]. However, an access-control-based method has several limitations. It only gives a “yes” or “no” option for the use of images, whereas parts of the information in images need to be used by applications such as Google Street View, and as it cannot automate privacy protection based on the privacy information of the image itself, it requires human participation.

More recent image privacy research focuses on the inherent implicit information of the photos. Tonge et al. [10] explore learning models that can automatically classify the private or public parts in an image using deep neural networks. Yu et al. [11] create a new tool called “iPrivacy” which uses a deep learning algorithm to detect privacy-sensitive objects. Yu’s work can detect the private parts of photos, but in the privacy protection step, they only use blur to protect privacy which does not look good. Uittenbogaard’s work [12] goes one step further than blurring and sets a framework that automatically removes moving objects. However, there are two limitations, one is that it is only for moving objects and the other limitation is that it missed partial information of the image. Liu’s work [13] proposes a novel Stealth algorithm, which prevents an automatic detector from detecting the objects in an image. However, humans can easily extract private information from an image.

Our framework is a further advancement compared with the aforementioned research. It can identify the private parts of photos at the pixel level. Then, it generates the target replacement content based on the private content, not just using a mosaic, blurring or removal to protect privacy. Our framework can protect private information from both humans and machines.

### 2.2. Deep-Learning-Based Image Object Detection and Segmentation

Object detection and semantic segmentation technologies have been advancing rapidly in recent years. In the beginning, Girshick et al. [21] used high-capacity convolutional neural networks (CNNs) for bottom-up region proposals, called R-CNN. This algorithm improves the mean average precision (mAP). In 2015, Hariharan et al. [22] defined the hypercolumn at a pixel as the vector of activations of all CNN units above that pixel to improve the results of the experiment. After this, of the majority of the research is based on the Fast R-CNN [23,24] and Fully Convolution Network (FCN) [25]. The disadvantage of Faster R-CNN is that it cannot deal with pixel-to-pixel alignment between the inputs and outputs of the network. To solve this problem, He et al. proposed a method called Mask R-CNN [26] that extends the Fast R-CNN by adding prediction segmentation masks on each region of interest (RoI) to get the results. As our goal is to find the private parts of images, we use Mask R-CNN to obtain the instance segmentation results that can be used as the basis for the follow-up privacy content detection and positioning. To obtain good results for our use case, we need to re-train the network using our image dataset which includes more privacy-sensitive content.

### 2.3. GAN-Based Content Generation

Preliminary ways to perform image privacy content protection include blurring, deletion, etc. In this paper, we replace content to protect privacy, i.e., generating content without any identifying information to replace the private content in the images. Traditional content generation methods such as [27,28,29,30] merely fill the pixels by matching and pasting based on the low-level features in the images. The effect is not very satisfactory as they often produce error content and the results obtained are also not good. In 2014, Goodfellow proposed a new framework called GAN [31] which can synthesize new content by training the models. Following the GAN-based method, the latest GAN-based generation content generation technology can generate very realistic content, such as faces, cats, dogs, even Airbnb rooms [32,33,34,35]. In our framework, we use StyleGAN [36] to generate the replacement content. StyleGAN can generate content which is not much different from the real image. The image content generated by StyleGAN does not exist in real life and this content can avoid copyright disputes. With the replacement of the generated content, the privacy of the images can be protected.

### 2.4. Privacy Protection

Privacy protection is an essential component for information system and management, such as network security [37,38], reputation management [39], blockchains [40] and the next generation of communication systems [41]. In traditional privacy protection technology, one of the most common methods is data encryption, which has high security. However, directly encrypting and decrypting large-scale data such as image sets will consume a lot of computing resources. Another privacy protection method is anonymity privacy protection technology. In 2002, Sweeney et al. proposed the k-Anonymity [42] method to protect privacy. Machanavajjhala proposed l-Diversity [43] to address the limitations of k-Anonymity, and Li et al. introduced t-Closeness [44]. However, with the development of attack technology, attackers can use data mining, machine learning, background knowledge attack, and big data analysis to obtain enough useful information on a user’s privacy. To solve this problem, Dwork [45] proposed the concept of differential privacy which has a solid mathematical theoretical foundation. Once differential privacy was proposed, it attracted attention in the field of privacy protection, and various privacy protection algorithms based on differential privacy have been proposed. In this paper, we propose a new image privacy protection method based on the differential privacy method combined with GANs. Taking advantage of the controllability of differential privacy, our method can protect the privacy of IoMT images with high controllability.

## 3. Preliminaries

In this section, we discuss image privacy protection, image utility, provide the basic knowledge and equations definition of our method.

### 3.1. Privacy Protection and Image Utility

**Figure 2:**
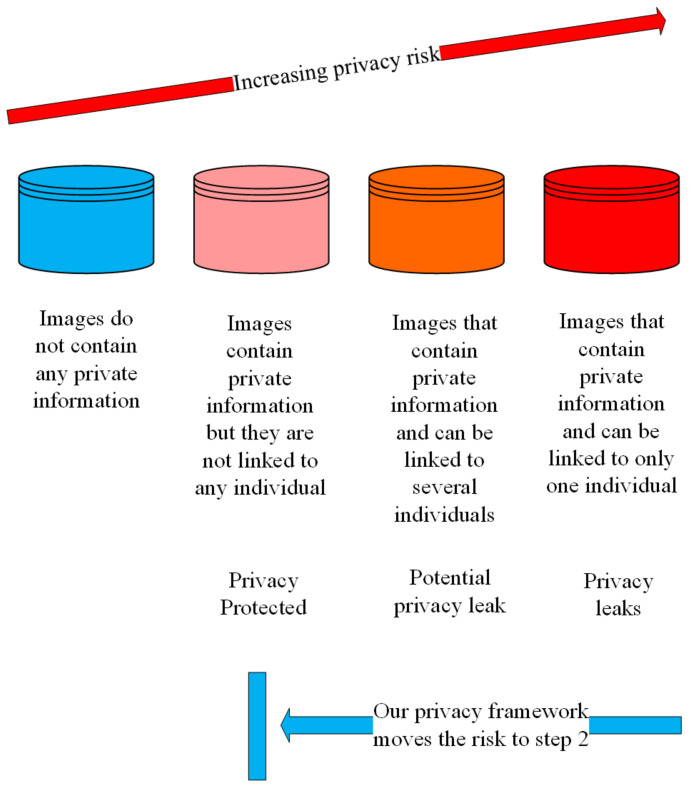
The four levels of image privacy risks.

In this part, we discuss image privacy protection and image utility. Firstly, the different levels of image privacy risk are shown in Figure 2. On the left are the images that do not contain any private information (such as a landscape photograph) and the risk of privacy leakage is zero. On the right are the images that contain private information which can be linked to specific individuals which violates individuals’ privacy directly. Between the two extreme cases are images that contain private information but might not leak individuals privacy. Our goal is to propose a framework to reduce the risk of privacy leak from Level 3/4 back to Level 2 in Figure 2. This means that we can protect the private information in images so that they cannot be linked to any individual.

**Figure 3:**
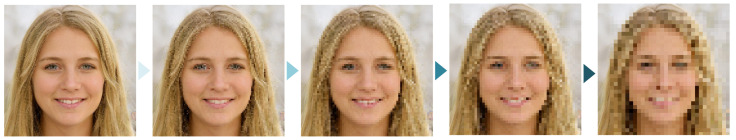
The privacy and utility.

However, the strength of privacy protection will affect the utility of images. The common methods such as mosaic and blur, might reduce the utility of the image while image processing. Greater privacy protection, results in lower utility of images, as shown in the example in Figure 3. Although the mosaic and blur methods protect privacy, it reduces the readability and usability of the images. It also makes image sharing pointless. In our image privacy protection framework, we have developed an effective way to compromise between privacy protection and image utility.

### 3.2. Formulation of Image De-Identification

We now formally define the problem of image de-identification which enables us to define the problem we need to address and build the foundation for the following discussions.

**Definition 1 (*****Image*****).**  *An image is a matrix I of m columns, n rows and c channels. There are usually 3 channels in the common color space, such as RGB and YUV. Each cell in matrix I contains a coding which ranges from 0 to 255. Images should contain multiprivate objects such as face or text.*

**Definition 2 (*****Object sets*****).**  *An object set is a set of M object images contained in image matrix I: $O_{i}:i=1,2,\ldots ,M$.*

**Definition 3 (*****Private object sets*****).**  *A private object set is a set of N objects images contained in image matrix I: $P_{i}:i=1,2,\ldots ,N$ in which $P_{i}\in O_{i}$ and N≤M.*

**Definition 4 (*****Private Object De-Identification Function*****).**  *Let* P *and* $P_{d}$ *be a private object set and a de-identification object set.*

(1) $$g:P\overset{}{\to }P_{d}$$

*where g is defined as the de-identification function for each $P_{i}$ to remove their identity.*

**Definition 5 (*****Image De-Identification*****).**  *Given image matrix I and de-identification function g, for each private object $P_{i}\in O_{i}$:*

(2) $$I_{d}=g\left(I\right)$$

*in which we can use the de-identification function to get an image matrix $I_{d}$ with no private information.*

### 3.3. Differential Privacy

**Definition 6 (*****Differential Privacy*****).**  *The formal definition of DP is given by Equation (3):*

(3) $$Pr\left[K\left(D_{1}\right)\in S\right]\leq exp\left(\epsilon \right)\times Pr\left[K\left(D_{2}\right)\in S\right]$$

**Definition 7 (*****The Sensitivity of Differential Privacy*****).**  *The sensitivity of DP is defined in Equation (4), which determines how much perturbation is required in the DP mechanism.*

(4) $$\Delta f=\max_{D_{1},D_{2}}{||f\left(D_{1}\right)-f\left(D_{2}\right)\left|\right|}_{1}$$

## 4. Image De-Identification Framework

In order to achieve the goal of image privacy protection, we propose an image de-identification framework which comprises three steps: (a) object detection and private object extraction; (b) de-identification content generation; and (c) content replacement and image privacy protection.

**Figure 4:**
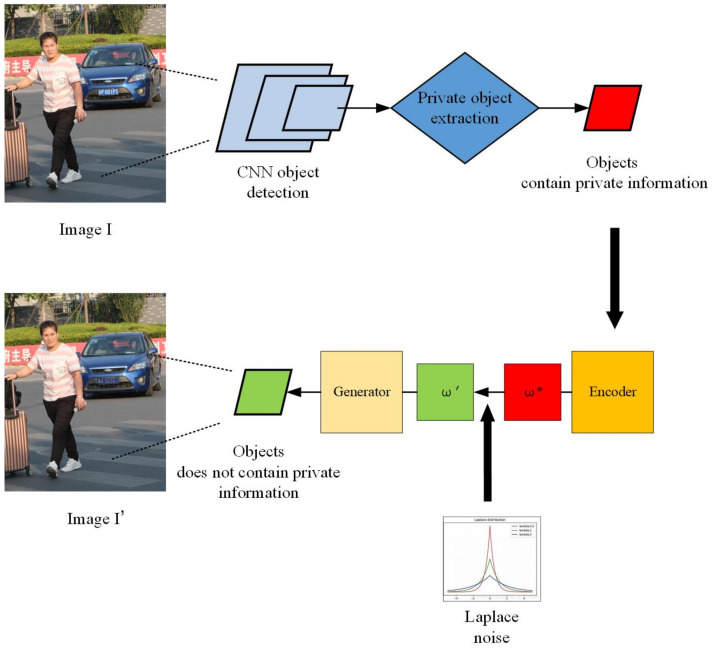
The diagram of the proposed image de-identification (DE-ID) framework.

Figure 4 shows the diagram of the framework. The original image *I* contains private information such as a face or a car license plate. It is first input into a CNN to identify and extract the private objects in the image. Then we transform the extracted private objects into latent space and use differential privacy to control the de-identified content generation. Finally, we obtain a de-identified image $I^{\prime }$, i.e., an image without any sensitive information. In the following part of this section, we explain the framework in detail.

### 4.1. Step-I: Object Detection and Private Object Extraction

To protect the privacy of an image, it is necessary to detect the sensitive privacy zones in the image. We use two steps to achieve this. First, all the objects in the image are detected, and then the private objects are extracted.

#### 4.1.1. Object Detection

**Figure 5:**
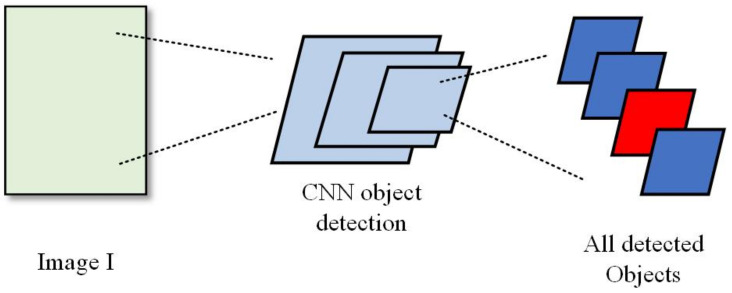
The diagram of object detection part in our image de-identification (DE-ID) framework.

The state-of-the-art object detection algorithm Mask-RCNN is used to detect the objects in the image. The diagram of object detection is shown in Figure 5. Images contain private information are detected by object detection algorithm Mask-RCNN, and the Mask-RCNN can detect all objects and position of the Images.

For an image *I*, the ROI vector $X_{roi}$ of each object $O_{i}$ can be detected by R(·):

$$X_{roi}=R\left(I\right)=(S|E)$$

(5) $$=\left(\begin{array}{cccccccc} x_{1} & y_{1} & w_{1} & h_{1} & E_{11} & E_{12} & \cdots & E_{1m}\\ x_{2} & y_{2} & w_{2} & h_{2} & E_{21} & E_{22} & \cdots & E_{2m}\\ \vdots & \vdots & \vdots & \vdots & \vdots & \vdots & \vdots & \vdots \\ x_{n} & y_{n} & w_{n} & h_{n} & E_{n1} & E_{n2} & \cdots & E_{nm} \end{array}\right)$$

where $S_{n}=\left(x_{n},y_{n},w_{n},h_{n}\right)$ is the position vector including the information of the upper left corner coordinate $\left(x_{i},y_{i}\right)$, width $w_{i}$ and height $h_{i}$ of object $O_{i}$. The probability of objects noted as E, the $E_{i}$ is the probability of Object $O_{i}$ belonging to the *m*th class (there are *m* class objects in the image *I*).

In Equation (5), we choose the maximum probability $c_{i}$ in each $E_{i}$, so the output of the object detection is shown as follows:

(6) $$O=\left(S|C\right)=\left(\begin{array}{ccccc} x_{1} & y_{1} & w_{1} & h_{1} & c_{1}\\ x_{2} & y_{2} & w_{2} & h_{2} & c_{2}\\ \vdots & \vdots & \vdots & \vdots & \vdots \\ x_{n} & y_{n} & w_{n} & h_{n} & c_{n} \end{array}\right)$$

where $\forall i\in \left(1,n\right):\;c_{i}=\left\{\begin{array}{c} \arg max\left(E_{ij}\right),1\leq j\leq m;\mathrm{if}\;max\left(E_{ij}\right)>\delta \\ c_{bg},\mathrm{if}\;max\left(E_{ij}\right)\leq \delta \end{array}\right.$.

In Mask-RCNN, if the maximum probability is smaller than a threshold δ, this object will be treated as the background class, otherwise the object belongs to class *i*.

#### 4.1.2. Private Objects Extraction

**Figure 6:**
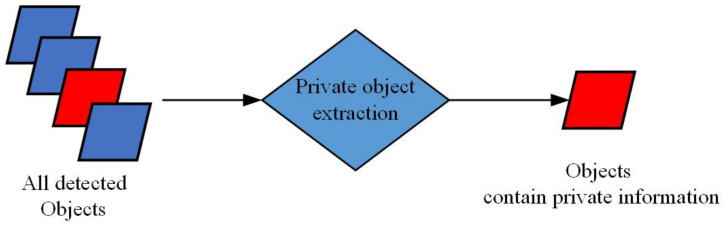
The diagram of private objects extraction part in our image de-identification (DE-ID) framework.

After obtaining the objects’ information and position, we set a classifier to classify the objects as either private or non-private. Figure 6 is the diagram of private objects extraction part in our framework. As shown in Figure 6, we extract the private objects from all detected objects. In the street view experiment, the private objects can be human faces, car license plates, etc. The non-private objects can be the background, trees, traffic lights.

The extraction process is finished by T(·) accordingly as shown in Equation (7).

$$T\left(O\right)=T\left(\begin{array}{ccccc} x_{p1} & y_{p1} & w_{p1} & h_{p1} & c_{p1}\\ \vdots & \vdots & \vdots & \vdots & \vdots \\ x_{p\alpha } & y_{p\alpha } & w_{p\alpha } & h_{p\alpha } & c_{p\alpha }\\ x_{np1} & y_{np1} & w_{np1} & h_{np1} & c_{np1}\\ \vdots & \vdots & \vdots & \vdots & \vdots \\ x_{np\beta } & y_{np\beta } & w_{np\beta } & h_{np\beta } & c_{np\beta } \end{array}\right)$$

(7) $$\;\;\;\;\;\;\;\;\;\;\;\;\;\;=\left(\begin{array}{ccccc} x_{p1} & y_{p1} & w_{p1} & h_{p1} & c_{p1}\\ x_{p2} & y_{p2} & w_{p2} & h_{p2} & c_{p2}\\ \vdots & \vdots & \vdots & \vdots & \vdots \\ x_{p\alpha } & y_{p\alpha } & w_{p\alpha } & h_{p\alpha } & c_{p\alpha } \end{array}\right)$$

where $\left(x_{pi},y_{pi},w_{pi},h_{pi}\right)$ and $c_{pi},i=1,\ldots ,\alpha$ are the position and class of private objects, and $\left(x_{npj},y_{npj},w_{npj},h_{npj}\right)$ and $c_{npj},j=1,\ldots ,\beta$ are the position and class of non-private objects. So we obtained the private objects’ position, class, and pixel information.

The private objects’ information is represented as follows:

(8) $$P=T\left(S|C_{p}\right)=\left(\begin{array}{ccccc} x_{p1} & y_{p1} & w_{p1} & h_{p1} & c_{p1}\\ x_{p2} & y_{p2} & w_{p2} & h_{p2} & c_{p2}\\ \vdots & \vdots & \vdots & \vdots & \vdots \\ x_{p\alpha } & y_{p\alpha } & w_{p\alpha } & h_{p\alpha } & c_{p\alpha } \end{array}\right)$$

### 4.2. STEP-II: De-Identification Content Generation

In the second step, we use a content generator G(·) and the differential privacy method to generate the de-identified content. The Algorithm 1 is shown as follows:
**Algorithm 1:** Image de-identification content generation.**  Input:** The private image $I_{p}\in {ℜ}^{n\times m\times 3}$ to de-identify; A pretrained generator   G(·).

**Figure 7:**
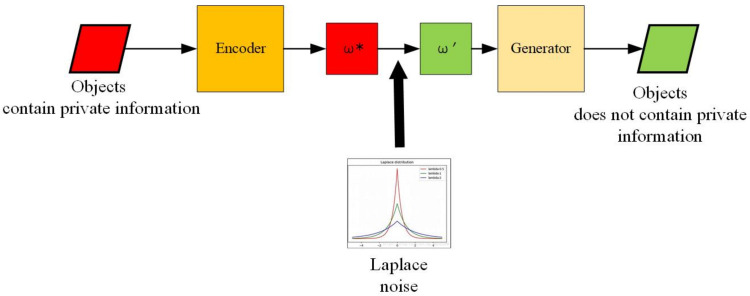
The diagram of de-identification content generation part in our image de-identification (DE-ID) framework.

**  Output:** The de-identified image $I_{d}$ optimized via G(·)  Initialize latent vector ω, differential privacy Laplace noise with Δf and ϵ;
**  while**
*not converged*
**do**
$\;\;|\;\;I_{p}≃I_{p}^{\prime }=G\left(\omega^{*}\right)$;
**  end**(
$\;\;I_{pd}=G\left(\omega^{*}+Lap\left(\frac{\Delta f}{\epsilon }\right)\right)$ ;


Figure 7 is the diagram of de-identification content generation part in our framework. Firstly, we find the latent vector $\omega^{*}$ of each input image $I_{p}$ which contains private information. We initialize a latent vector ω and search for an optimized vector $\omega^{*}$ which minimizes the loss function (9). This loss function measures the similarity between the private object image and an image generated by latent vector $\omega^{*}$ [46].

(9) $$\omega^{*}=\overset{min}{\underset{\omega }{}}L_{percept}\left(G\left(\omega \right),I_{p}\right)+\frac{\lambda_{mse}}{N}||G\left(\omega \right)-I_{p}{\left|\right|}_{2}^{2}$$

where image $I_{p}\in {ℜ}^{n\times m\times 3}$ is the input private image. G(·) is the pretrained generator, N is the number of scalars in the image, ω is the latent code to optimize, $\lambda_{mse}=1$.

The loss term $L_{percept}$ is calculated as follows:

(10) $$L_{percept}\left(I_{p1},I_{p2}\right)=\sum_{j=1}^{4}\frac{\lambda_{j}}{N_{j}}\left|\right|F_{j}\left(I_{p1}\right)-F_{j}\left(I_{p2}\right){\left|\right|}_{2}^{2},$$

where $I_{p1},I_{p2}\in {ℜ}^{n\times m\times 3}$ are the input private images, $F_{j}$ is the feature output of VGG-16 layers conv1_1, conv1_2, conv3_2, conv4_2. $N_{j}$ is the number of scalars in the *j*th layer output, $\lambda_{j}=1$ for all *j*s are empirically obtained for good performance.

Above step enables the image to be editable. Secondly, after we obtain latent vector $\omega^{*}$ of each private object, we put the Laplace noise on latent vector $\omega^{*}$ and get the new latent vector $\omega^{\prime }$.

(11) $$\omega^{\prime }=\omega^{*}+Lap\left(\frac{\Delta f}{\epsilon }\right)$$

Then, we put the new latent vector $\omega^{\prime }$ into the generator G(·) and obtain the de-identified content $I_{pd}$.

(12) $$I_{pd}=G\left(\omega^{*}+Lap\left(\frac{\Delta f}{\epsilon }\right)\right)$$

In Equation (12), we used the DP criterion to protect the sensitive information in an image using the Laplace mechanism. Generally speaking, the Laplace mechanism adds a controlled Laplace noise to a query result before returning it to the user. Here, the Laplace noise is sampled from a Laplace distribution, which is shown in Equation (13).

(13) $$Lap\left(x\right)=\frac{1}{2b}exp\left(-\frac{\left|x\right|}{b}\right)$$

The Laplace mechanism can be summarized as

(14) $$M\left(D\right)=f\left(D\right)+Lap\left(\frac{▵f}{\epsilon }\right)$$

The Laplace mechanism in Equation (14) indicates that the size of the Laplace noise is related to the sensitivity of query *f* and the privacy budget ϵ. A larger sensitivity leads to a higher noise. In our method, we use privacy budget ϵ to control our GAN generator to generate the synthetic de-identified content.

### 4.3. STEP-III: De-Identified Content Replacement

After de-identified content has been generated, we use the generated content to replace the original private object images. The Algorithm 2 is shown as follows:
**Algorithm 2:** Image protected by de-identification content swapping.**  Input:** The original image $I\in {ℜ}^{n\times m\times 3}$ contains private objects $\;\;P^{i},i=1,2,\ldots ,N$;
  de-identified objects in the image: $P_{d}^{i},i=1,2,\ldots ,N$
**  Output:** The protected image $I_{d}\in {ℜ}^{n\times m\times 3}$
**  for**
*each $P_{d}^{i}$*
*** in **$P_{d}$*
**do**
$\;\;\;|\;\;P^{i}\overset{swapping}{⟵}P_{d}^{i}$

**  end**
$\;\;I_{d}=g\left(I\right)$


**Figure 8:**
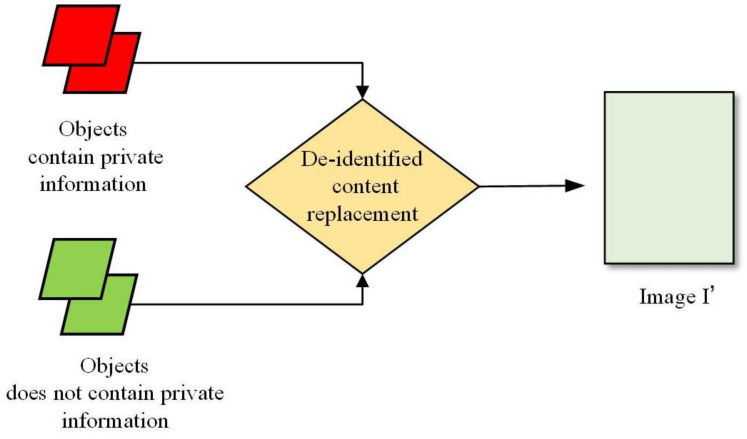
The diagram of de-identified content replacement part in our image de-identification (DE-ID) framework.

The original image *I* contain private objects and we use the de-identified objects generated by our method to replace the private objects. Figure 8 is the diagram of de-identified content replacement part in our framework. We use the images of the de-identify objects (not contain private information) to replace the original private object images (contain private information). After the private image *I* processed by the above three steps, we finally obtain the de-identified image $I_{d}$.

## 5. Experiments and Discussions

In this section, we provide the experiment setup, performance evaluation metrics, street view image protection (include human face privacy protection and car license plate privacy protection) and performance evaluation.

### 5.1. Experiment Setup

First of all, we set up an experiment database containing the street view images collected by IoMT technology. The street view images contain human faces, car license plates, road signs, traffic lights and more. In these images, the sensitive private information are human faces and car license plates. In our test database, the human faces and car plates are the private objects, and the road sign, the traffic light and background are the non-private objects. We use the camera to collect about 4000 typical street view images as the test database.

### 5.2. Performance Evaluation Metrics

#### 5.2.1. Privacy Metrics

**Confidence Score**. In the privacy protection metric for a human face, we use the open-source “face recognition” platform to evaluate the confidence in face privacy. This platform was built using dlib’s state-of-the-art face recognition which was built with deep learning. The model has an accuracy of 99.38% on the Labeled Faces in the Wild benchmark. The output of the platform is the facial distance between each unrecognized face and the recognized face. By setting the corresponding threshold, the distance metric can judge whether the face is protected. This means after the face photo is processed by our method, we can determine whether the general third-party platform still considers it be the same person. The default threshold is 0.3.

**Distance**. In the privacy protection metric for the car license plate, as the license plate is a set of characters, we believe that the distance between the original license plate and the processed license plate is the privacy metric. In the experiment, we set the threshold of the car license plate as 3. This means that the sensitive information on the license plates is protected when the distance is greater than 3.

#### 5.2.2. Image Utility Metrics

Quantitative judgment is necessary for the degree of modification between the original image and the protected image. So, we use several metrics to calculate the degree of modification, including $L_{0}$, $L_{2}$, $ALD_{p}$, the structural similarity index (SSIM), and the difference value hash(Dhash). Deciding when there are two images: processed image $I^{a}$ and original image *I*, the utility image metrics are:

$L_{0}$ calculates the number of changed pixels.

(15) $$L_{0}=num\left(I^{a},I\right)$$

where num is calculated as the number of pixels changed between $I^{a}$ and *I*.

$L_{2}$ calculates the Euclidean distance between the original image and the protected image.

(16) $$L_{2}=\left|\right|I^{a}-I{\left|\right|}_{2}=\sqrt{\sum_{i=1}^{N}{\left(I_{i}^{a}-I_{i}\right)}^{2}}$$

ALD calculates the average *L* distance between the images.

(17) $$ALD_{p}=\frac{1}{n}\sum_{i=1}^{n}\frac{∥I_{i}^{a}-I_{i}{∥}_{p}}{∥I_{i}{∥}_{p}}$$

SSIM is the common method to evaluate the similarity between the original image and the protected image.

(18) $$SSIM\left(I^{a},I\right)=\frac{1}{n}\sum_{i=1}^{n}SSIM\left(I_{i}^{a},I_{i}\right)$$

Dhash uses the difference hash to evaluate the degree of modification where the smaller the value, the better.

(19) $$Dhash\left(I^{a},I\right)=hash\left(I^{a}\right)-hash\left(I\right)$$

### 5.3. Street View Image Protection

#### 5.3.1. Human Face Privacy Protection

The human face is the most sensitive information in IoMT images, which can reveal a person’s identity. Therefore, we use our method to protect facial privacy in the street view experiment.

The algorithm to de-identify the facial image is shown in Algorithm 3.
**Algorithm 3:** Facial image de-identification.**  Input:** A human face image $I\in {ℜ}^{n\times m\times 3}$ to protect; a pretrained generator G(·)
**  Output:** The de-identify facial image $I_{d}$. 
  Initialize latent code $\omega^{*}=\omega$; 
**  while**
*not converged*
**do**
$\;\;\;|\;\;I≃I^{\prime }=G\left(\omega^{*}\right)$;
**  end**(
$\;\;I_{d}=G(\omega^{*}+Lap(\frac{\Delta f}{\epsilon }))$


Firstly, we use Mask-RCNN to extract facial images *I* from the experiment street view images.

Secondly, we initialize latent vector ω and use the loss function to find latent vector $\omega^{*}$ of human face *I*.

Thirdly, we put the Laplace noise on latent vector $\omega^{*}$ and use generator G(·) to generate the de-identified facial image.

(20) $$I_{d}=G\left(\omega^{*}+Lap\left(\frac{\Delta f}{\epsilon }\right)\right)$$

Finally, we swap the de-identified facial image for the original facial image. In this step, we use Dlib, which is a toolbox in OpenCV based on key-point face detection, to obtain the 68 key points of the faces and use seamless cloning to swap the faces. The face swapping algorithm can transfer the input facial features to the target face without being obtrusive.

**Figure 9:**
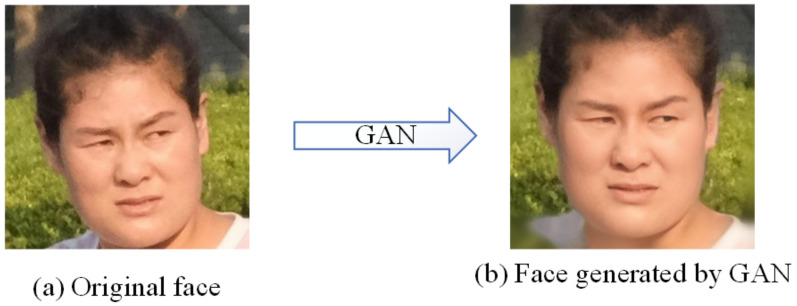
The original face image projected into StyleGAN: (**a**) Original face. (**b**) Face generated by GAN.

Figure 9 is an example of the original human face image and the human face generated by GAN with no modification.

**Figure 10:**
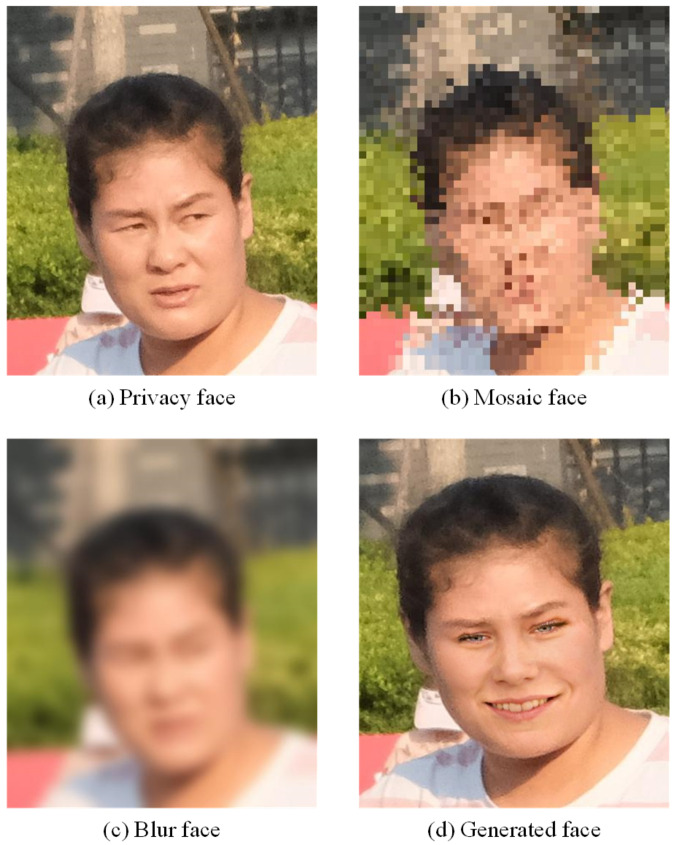
Face images comparison: (**a**) face in street view, (**b**) mosaic methods, (**c**) blur method, and (**d**) our method.

The de-identified example result is shown in Figure 10. Intuitively speaking, a larger Laplace noise results in the generation of a very different face compared with the original photo of the face. In our experiments, we use Laplace noise parameter ϵ to control the distance between the de-identified facial image and original facial image. In addition, we use the open-source “face recognition” platform to determine if the synthetic facial image and the original facial image represent the same person.

#### 5.3.2. Facial Privacy Protection Discussion

There are currently many methods for face swap and generation, the main methods include DeepFakes [47], Face2Face [48], FaceSwap [49], and NeuralTextures [50], etc. These methods can well swap the source face to achieve the purpose of changing the source face. However, in 2019, Andreas et al. propose an automated benchmark for facial manipulation detection called FaceForensics++ [51] which can detect manipulated facial images. This benchmark can easily detect the manipulated facial images with high efficiency. However, the previous methods discussed the application and effects of face swapping and how to detect manipulated facial images. We are more concerned about the privacy protection of IoMT images. In our method, we propose a framework that uses GAN and DP to protect the multiobject privacy of IoMT images. Different from other face swap methods, our method can not only protect facial information but also protect other private information, such as license plates. Our replacement content is generated by GAN, and DP technology is applied to control the generated content.

Figure 10 shows the original photo of the face, the mosaic face, the blurred face, and the new facial image generated by our method. It can be seen that it is not easy for either a human or a machine to recognize the de-identified generated facial image in Figure 10d compared to Figure 10a. It is worth mentioning that if the protected person sees that his∖her facial information is replaced by computer-generated content, he should feel at ease because his∖her personal facial information has been de-identified.

To protect the privacy of IoMT images, just replacing faces is not enough to protect privacy, multiobject privacy needs to be protected. So we set a framework that can protect multiobject privacy. There are many private contents in IoMT images that need to be protected. In our method, we added car license plates as another type of privacy that needs to be protected.

#### 5.3.3. Car License Plate Privacy Protection

Car license plates are another type of sensitive objects in IoMT images. We use Chinese car license plates as our experimental objects. The car license plates should be generated according to the rules enforced by the vehicle management authority. The rules of a valid Chinese car license plate are: (1) the first character is a Chinese character, representing a province; (2) the second symbol is an English letter; (3) the last five symbols form a random string of letters and numbers, and (4) the background of a license plate is dark blue.

After obtaining the car license plate images from the street images, we use OCR to recognize the characters and symbols on the car license plates, and then map the car license plate into a sequence of numbers. As previously mentioned, the first character will be one of 31 Chinese province abbreviation characters (with the exception of special districts). Because the first Chinese character represents location information, we map this into two-digit numbers 00–30 based on the sorted distances from each province to the capital city Beijing. The mapping table for the first character is shown in Table A1.

**Figure 11:**
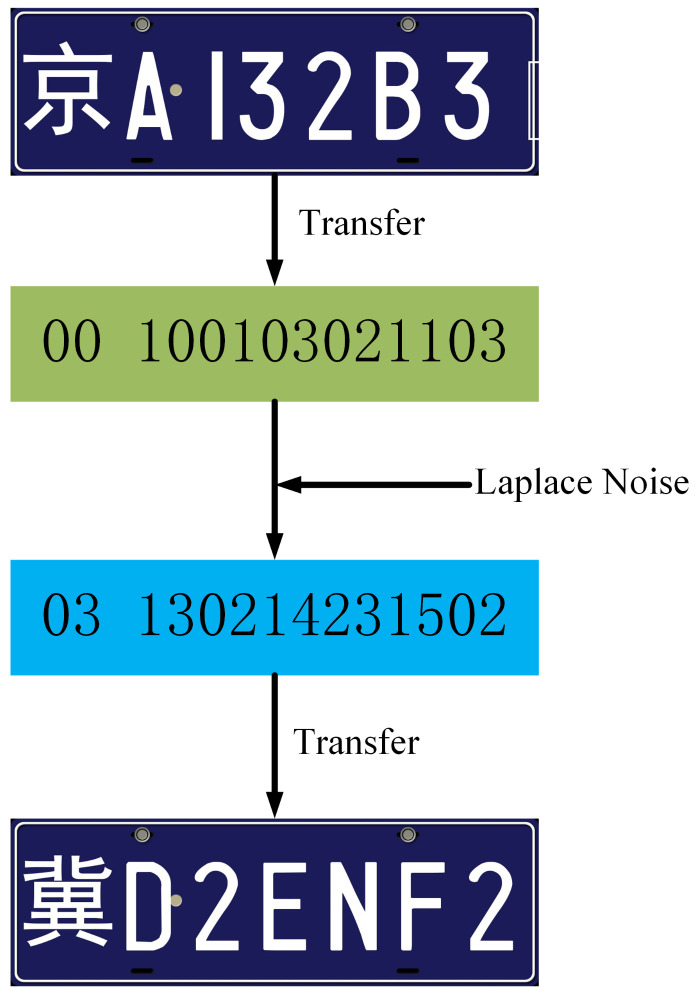
A new car plate content created by DP.

Next, the numerical values 0–9 are translated into two-digit codes 00–09, and the English symbols are translated into two-digit codes 10–33. For example, a car plate “Beijing A132B3” will be mapped to a sequence of numbers “00 100103021103”. After we translate each car plate into a sequence of numbers, we add Laplace noise onto the number sequence and obtain a synthetic number sequence satisfying DP. In Laplace noise generation, we let Δf=1 and control ϵ to generate the Laplace noise. For example, if we add a random Laplace noise on the above car plate “00 100103021103”, we obtain a perturbed sequence as “03 130214231502”, which can be translated to a synthetic car plate “Hebei D2ENF2”. The above example is illustrated in Figure 11. There is a cyclic shift if the Laplace noise results in the value being out of the bounds, e.g., the province code > 33.

Then, we use the generator to generate a synthetic car plate image according to the car plate code. Finally, we swap the car plate with the synthetic car plate image. The synthetic car plate is protected by the DP criterion.

In the car license plate number transfer, the larger the noise, the longer distance car number is generated. For example, if a province name is Jilin on a car plate, the province codes should be generated for Jilin based on the distance from the other provinces to Jilin.

#### 5.3.4. Car License Plate Privacy Protection Discussion

Car license plates as another type of sensitive objects in IoMT images. We choose car license plate as another type of multiobject privacy protection representative. The framework we proposed can add more types of private objects, which is highly scalable. In future work, We will add more types of private objects.

It is important to note that the replacement of the private content in an image is not simply a copy-and-paste job. Instead, it needs to transform the synthetic content by generating an image that fits into the original image area with the correct orientation. Therefore, the synthetic image is generally not perceptible to human eyes.

**Figure 12:**
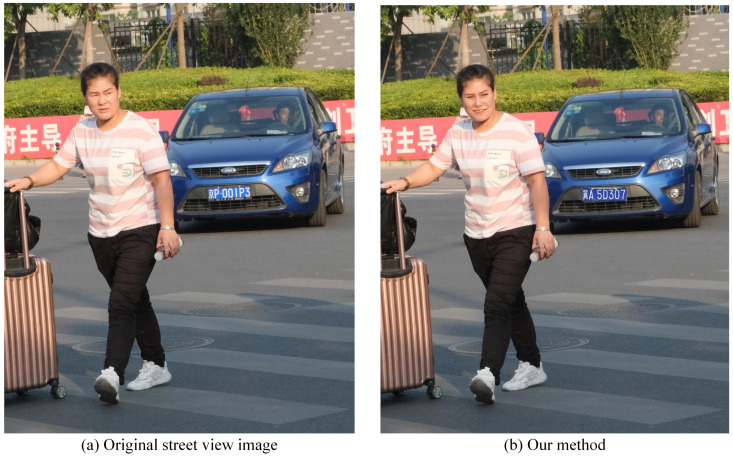
A typical Chinese car plate swap to protect the private information in street view image: (**a**) Original street view image. (**b**) Our method.

Our method uses the synthetic DP car plate to protect the private car plate information. As shown in Figure 12, we can see that the car plate is smoothly replaced by our synthetic car plate.

### 5.4. Performance Evaluation

#### 5.4.1. Privacy Protection Metrics

In this part, we calculate the distance between the original private image and the protected image to measure the degree of privacy protection.

For a human face, the average distance between the same person is 0.12, which has a confidence score of 88. After using our processing method, the average facial distance is 0.45 with a confidence score of 55, which is over the threshold of the confidence score of 70. This experiment result means our method can remove the identity of the human face, which means our method can protect the privacy of facial image.

For car license plates, because the license plates are strings, their distances are integers. In the experiment, the distance between the same license plate is 0. After using our processing method, the distance is 3, so the sensitive information in the image of the car license plate is protected.

#### 5.4.2. Image Utility Metrics

**Figure 13:**
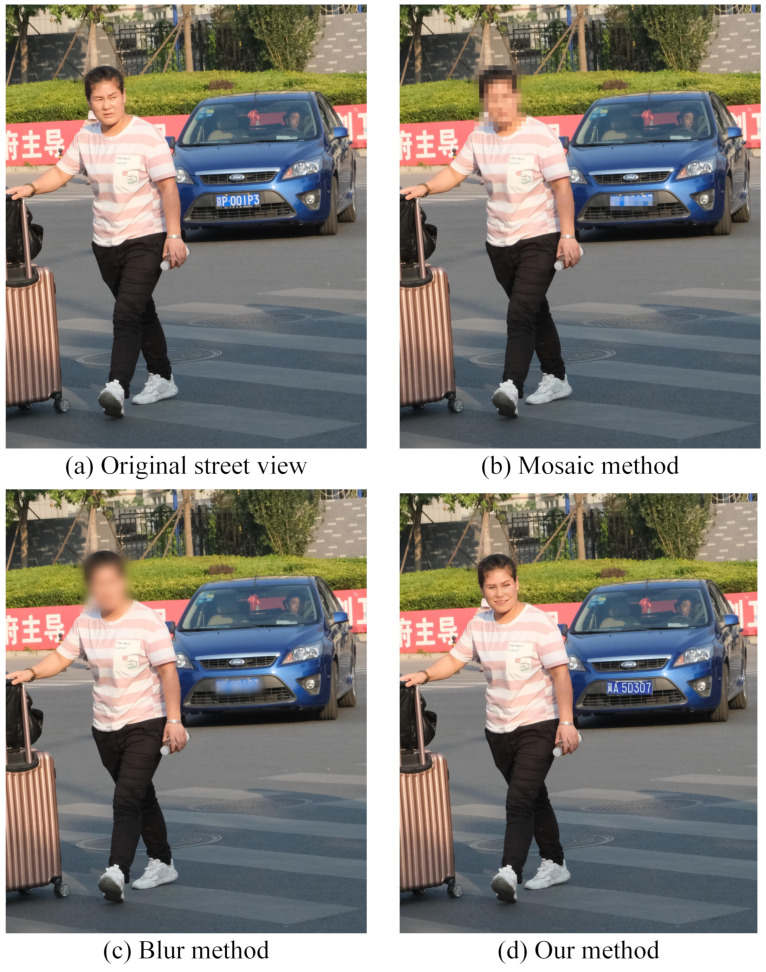
The result of four street view images: (**a**) unprotected image. (**b**) image processed with blur, (**c**) image processed with mosaic, (**d**) image processed with our method.

In this part, we set an automatic evaluation module to calculate the degree of image modification using different metrics through $L_{0}$, $L_{2}$, $ALD_{p}$, SSIM, and Dhash. We compare our method with the blur and mosaic methods. As shown in Figure 13, the blur and mosaic methods remove the sensitive private area. However, a human can easily notice the blur and mosaic in the image. Hence, a computer can recover the information from the processed image [52,53,54].

In our method, we control the generator to generate the de-identified content image with DP Laplace noise. The de-identified images make it very difficult for human eyes and computer vision detection methods to detect the differences and obtain the private information for sensitive private objects. The results of the street view image are shown in Figure 13. It can be seen that a human and a computer can easily detect the sensitive information in the unprotected street view image in Figure 13a. In Figure 13b,c, the algorithm cannot detect the face or the car license plate number after being blurred, but a human can easily see there is a blur or mosaic in the image. In relation to Figure 13d, both a computer algorithm and a human can detect the changed sensitive information, so neither a human nor a computer can not see the real sensitive information of the face and the car plate. Hence, our method protects the private information in the image.

Next, we use metrics to evaluate the efforts of our method. Table 1 shows the performance of our method, blur, and mosaic. The metrics are DHash, SSIM, $L_{0}$, $L_{2}$ and $ALD_{p}$. The blur and mosaic are modified to change the sensitive area in our experiment images.

First, compared with the other methods, our method changes the minimum number of pixels to protect the private part of the image. For Dhash, our method is better than the others. Compared with blur and mosaic, our method decreases the Dhash metric by 95.02% and 95.2%. Our method is better than the others in SSIM metric by 1.17% and 1.67%. Our method decreases the $L_{0}$ metric by 73.6% and 72.97% compare with blur and mosaic. In $L_{2}$ metric, our method decrease the $L_{2}$ metric by 86.25% and 25.99%. In $ALD_{p}$ metric, our method’s result is higher than blur and mosaic, which is 160.65% and 98.85%.

This shows that our method is better than the other two methods in metrics: SSIM, Dhash and $L_{0}$. However, the results show that in metrics: $L_{2}$ and $ALD_{p}$, our method is not the best. After analysis, we found that $L_{2}$ and $ALD_{p}$ are more suitable in big area modification. These metrics are not sensitive to minor modifications.

We use the facial image as an example to show the metrics’ results in the minor modification in a small area. We choose 4000 face swap images to analyze and the results are shown in Table 2. In Dhash metric results, compared with blur and mosaic, our method’s result decreases by 96.68% and 96.97%. In SSIM metric results, our method increases by 50.67% and 102.24% compared with blur and mosaic. In $L_{0}$ metric result, our method decreases by 76.55% and 76.84% than blur and mosaic. In $L_{2}$ metric result, our method decreases by 64.93% and 81.08% than blur and mosaic. In $ALD_{p}$ metric result, compared to blur and mosaic, our method decreases by 65.11% and 79.68%. As we can see, our method is the best for all metrics in the evaluation of minor modification in a small area.

## 6. Conclusions

In this paper, we propose a new image privacy protection method based on GAN and DP. Our method can protect the sensitive private information contained in IoMT images. We use the deep neural network to identify the private data in images and de-identify this with the GAN-based content. Compared with traditional blur and mosaic methods, the proposed method can protect the sensitive information in image data and avoid privacy leakage. The experiment results of the collected IoMT image data show that our privacy protection method can protect privacy with high efficiency and controllability. In future work, we will study the privacy protection of IoMT videos and improve the real-time nature of our method. We will also add more types of private objects into our framework and propose a higher effectively privacy protection method for the privacy of IoMT images.

## Figures and Tables

**Table 1:** Average result of 4000 street view images with the metrics: Dhash, SSIM, $L_{0}$, $L_{2}$, $ALD_{p}$.

Methods	Original	Blur	Mosaic	Our Methods
Dhash	0	12,873.65	13,370.19	641.71
SSIM(10${}^{-2}$)	100	98.18	97.70	99.33
$L_{0}$(10${}^{2}$)	0	1692.25	1652.57	446.74
$L_{2}$	0	9983.06	14,757.19	18,593.41
$ALD_{p}(10^{-2}$)	0	3.99	5.23	10.4

**Table 2:** Average result of 4000 facial images with the metrics: Dhash, SSIM, $L_{0}$, $L_{2}$, $ALD_{p}$.

Methods	Original	Blur	Mosaic	Our Methods
Dhash	0	4047.80	4427.79	134.25
SSIM(10${}^{-2}$)	100	64.63	48.15	97.38
$L_{0}$(10${}^{2}$)	0	1009.4	1022.25	236.72
$L_{2}$	0	5832.96	10,812.2	2045.48
$ALD_{p}(10^{-2}$)	0	16.68	28.64	5.82

## Data Availability

The data presented in this study are available on request from the corresponding author. The data are not publicly available due to privacy protection.

## Appendix A

**Table A1:** The transfer two-digit code based on distance between Beijing and each province of China.

Province Name	Distance to Beijing (km)	2-Digit Code
Beijing	0	00
Tianjin	96.07188	01
Hebei	239.4603	02
Shandong	356.9375	03
Shanxi	407.3106	04
Neimengu	424.5428	05
Henan	620.2232	06
Liaoning	630.724	07
Jiangsu	860.7032	08
Jilin	867.213	09
Ningxia	884.2019	10
Anhui	897.8403	11
Shanxi	907.8513	12
Hubei	1041.318	13
Shanghai	1041.987	14
Heilongjiang	1056.846	15
Zhejiang	1102.843	16
Gansu	1184.73	17
Jiangxi	1242.833	18
Hunan	1316.041	19
Qinghai	1340.82	20
Chongqing	1419.309	21
Sichuan	1505.931	22
Fujian	1527.525	23
Guizhou	1729.627	24
Guangdong	1856.641	25
Guangxi	2047.263	26
Yunnan	2068.306	27
Hainan	2249.545	28
Xinjiang	2433.955	29
Xizang	2559.149	30
